# Which is cheaper: a fully covered metallic stent or a choledochoscope?

**DOI:** 10.1055/a-2351-2645

**Published:** 2024-08-07

**Authors:** Liang-Hao Hu, Ping-Ping Zhang, Ting Yang, Yan-Wei Lv

**Affiliations:** 1 Department of Gastroenterology, Changhai Hospital, Naval Military Medical University, Shanghai, China

Benign biliary obstruction may occur in patients with chronic pancreatitis 1 2 . Endoscopic placement of a fully covered self-expandable metallic stent (FCSEMS) for biliary drainage is an effective treatment strategy for biliary obstruction 3 . However, the migration rate of FCSEMSs is approximately 10% to 33% 4 5 . We report successfully repositioning a dislocated biliary FCSEMS using a choledochoscope.

A 49-year-old man with chronic pancreatitis with benign bile duct stenosis was admitted due to obstructive jaundice. Endoscopic retrograde cholangiopancreatography (ERCP) was performed and a FCSEMS (EVO-FC, 6 cm; Cook Medical) was placed. The patient developed a fever on the 15th day after the operation. Abdominal computed tomography indicated migration of the biliary FCSEMS ( Fig. 1 ). Another ERCP procedure was performed. Cholangiography indicated that the FCSEMS in the common bile duct (CBD) had moved into the proximal bile duct and the stent was mobile ( Fig. 2 ). The lower segment of the CBD (a length of 2 cm) was significantly narrowed, while the diameter of the middle and upper segments of the CBD was dilated to approximately 1.3 cm. We used a choledochoscope (SpyGlass; Boston Scientific) to reposition the FCSEMS ( Fig. 3 ). The retrieval string of the FCSEMS was visible. A biopsy forceps (SpyBite Max; Boston Scientific) was inserted through the accessory biopsy channel of the choledochoscope ( Fig. 4 ) and the retrieval string was grasped under direct visualization. The displaced FCSEMS was partially pulled out to the opening of the duodenal papilla. Finally, the stent was fixed to the papilla opening with a hemostatic clip (ROCC-F-26-165C; Micro-Tech) to prevent it from moving again ( Fig. 5 , Video 1 ).

**Fig. 1:**
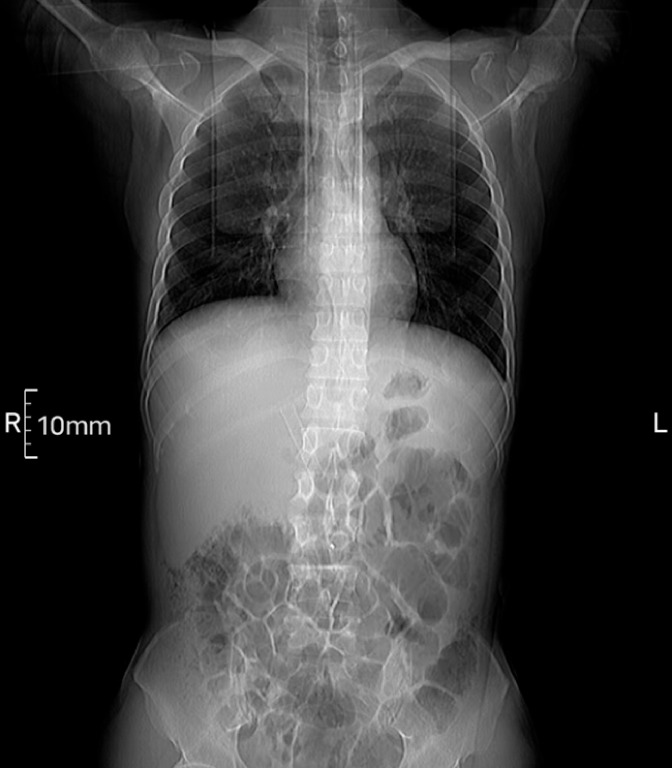
Abdominal coronal computed tomography showed migration of the biliary fully covered self-expandable metallic stent (FCSEMS).

**Fig. 2:**
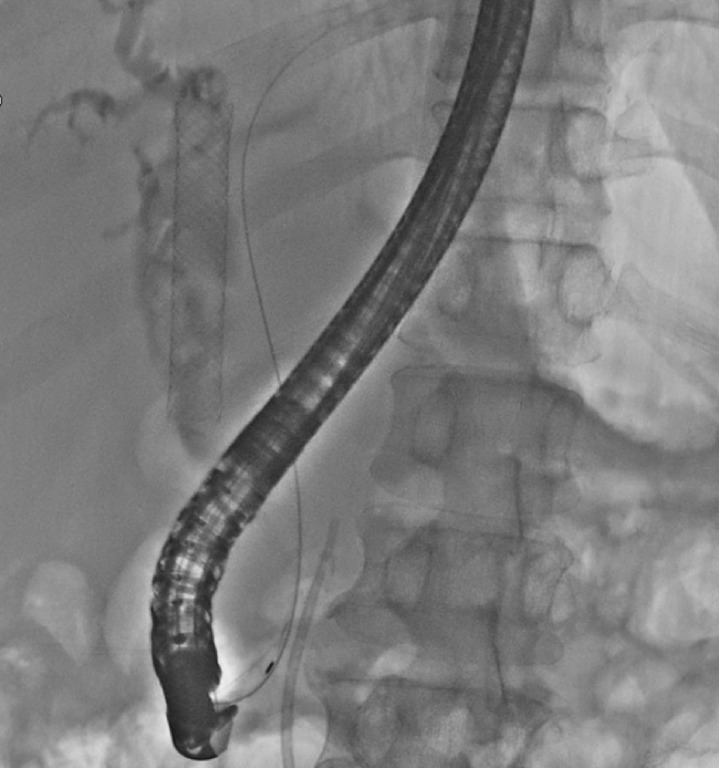
Cholangiography showed that the FCSEMS in the common bile duct had moved into the proximal bile duct.

**Fig. 3:**
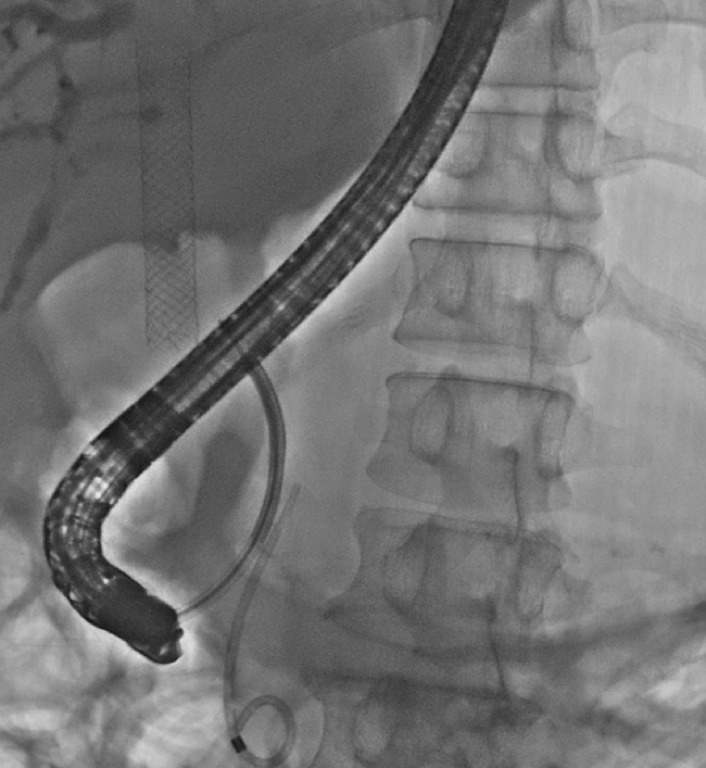
Use of a choledochoscope (SpyGlass; Boston Scientific) to directly visualize the distal end of the stent.

**Fig. 4:**
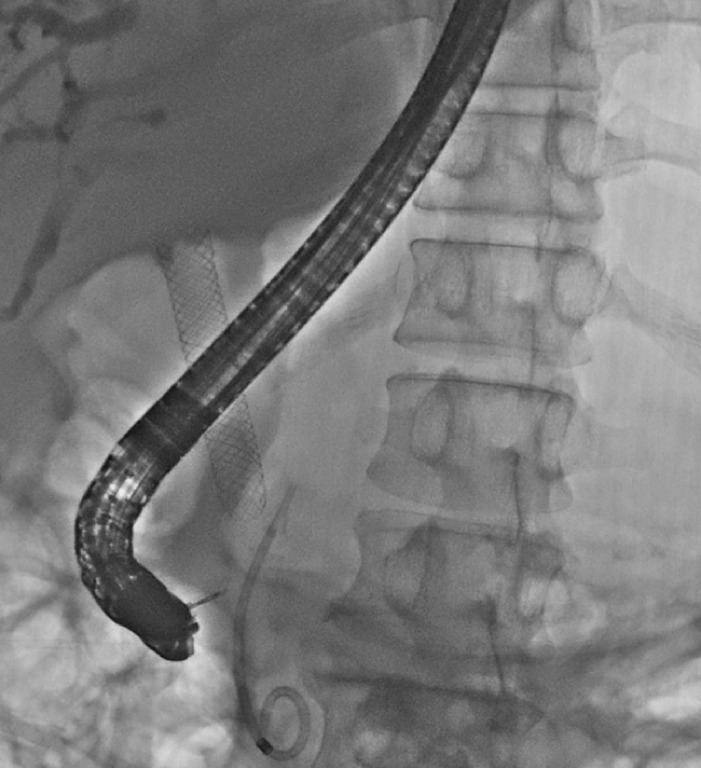
A biopsy forceps was inserted into the bile duct through the biopsy channel of the choledochoscope.

**Fig. 5:**
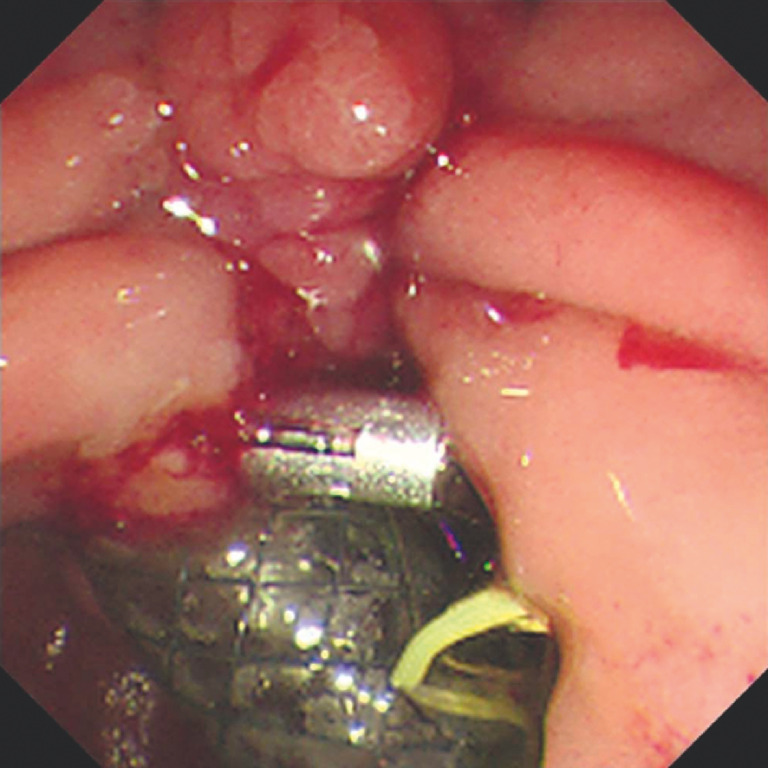
The stent was fixed to the surface of the papilla by means of a hemostatic clip.

Successful repositioning of a dislocated biliary fully covered self-expandable metallic stent using a choledochoscope.Video 1

Choledochoscope-assisted repositioning of biliary FCSEMSs can be considered as feasible and has the advantages of safety and easy operation. Repositioned stents are not deformed and can continue to be used. However, in clinical practice the therapeutic choice between using the choledochoscope to adjust a displaced FCSEMS or to replace the displaced stent with a new FCSEMS should be based on a comprehensive consideration of all the elements including local medical conditions and medical expenses.

Endoscopy_UCTN_Code_TTT_1AR_2AZ

**Correction: Which is cheaper: a fully covered metallic stent or a choledochoscope?**

**Hu Liang-Hao, Zhang Ping-Ping, Yang Ting et al. Which is cheaper: a fully covered metallic stent or a choledochoscope?** Endoscopy 2024; 56: E695–E696, doi:10.1055/a-2351-2645

In the above-mentioned article, the author name “Ting Yang” was corrected. This was corrected in the online version on August 22, 2024.

## Notes

Endoscopy E-Videos https://eref.thieme.de/e-videos
*E-Videos* is an open access online section of the journal *Endoscopy* , reporting on interesting cases and new techniques in gastroenterological endoscopy. All papers include a high-quality video and are published with a Creative Commons CC-BY license. Endoscopy E-Videos qualify for HINARI discounts and waivers and eligibility is automatically checked during the submission process. We grant 100% waivers to articles whose corresponding authors are based in Group A countries and 50% waivers to those who are based in Group B countries as classified by Research4Life (see: https://www.research4life.org/access/eligibility/ ). This section has its own submission website at https://mc.manuscriptcentral.com/e-videos .

## Notes

Correction: Which is cheaper: a fully covered metallic stent or a choledochoscope? **Hu Liang-Hao, Zhang Ping-Ping, Yang Ting et al. Which is cheaper: a fully covered metallic stent or a choledochoscope?** Endoscopy 2024; 56: E695–E696, doi:10.1055/a-2351-2645
In the above-mentioned article, the author name “Ting Yang” was corrected. This was corrected in the online version on August 22, 2024.
