# Correction: Which is cheaper: a fully covered metallic stent or a choledochoscope?

**DOI:** 10.1055/a-2392-2906

**Published:** 2024-08-22

**Authors:** Liang-Hao Hu, Ping-Ping Zhang, Ting Yang, Yan-Wei Lv

**Affiliations:** 1 Department of Gastroenterology, Changhai Hospital, Naval Military Medical University, Shanghai, China



In the above-mentioned article, the author name “Ting Yang” was corrected. This was corrected in the online version on August, 22 2024.

